# Behavioural assessment of the dysexecutive syndrome (BADS) in healthy elders and Alzheimer's disease patients: preliminary study

**DOI:** 10.1590/s1980-57642008dn10200007

**Published:** 2007

**Authors:** Fabiola Canali, Sonia Maria Dozzi Brucki, Orlando Francisco Amodeo Bueno

**Affiliations:** 1 Post graduate student, psychologist. Department of Psychobiology of Federal University of São Paulo, Brazil.; 2 MD, PhD. Department of Psychobiology of Federal University of São Paulo, Brazil.; 3 MsC, PhD, Head of Department. Department of Psychobiology of Federal University of São Paulo, Brazil.

**Keywords:** Alzheimer disease, executive functions, BADS, neuropsychology

## Abstract

Although the main initial deficit is considered to be in the memory domain, an early impairment of executive functions is also found in AD where these deficits are correlated to functional impairment. Ecological tests are more indicated to evaluate executive impairment, and are better able to assist in treating AD patients than more commonly used tests.

**Objectives:**

The aim of this preliminary study is to verify the performance in executive functions using the Behavioural Assessment of the Dysexecutive Syndrome (BADS) in elder controls and mild AD patients, and to analyze its applicability in our environment.

**Methods:**

The BADS was performed by 17 healthy elders and 17 early AD patients matched for age, schooling and gender.

**Results:**

There were significant differences among controls and AD patients on MMSE scores, and in measures of executive functions, memory, and motor speed. Some sub items of BADS (rule shift cards, modified six elements, total score, standard, standard by age and overall classification by age) were also different between groups. Differences were also significant on the Dysexecutive Questionnaire (DEX) of BADS self-ratings and other-ratings.

**Conclusion:**

BADS was efficacious in detecting executive deficits in this sample, as confirmed by other executive functions tests applied.

## Abstract (pt)

Embora o principal déficit inicial seja a memória, existe também um prejuízo nas funções executivas na DA desde as fases leves e estes estão correlacionados com prejuízo funcional. Testes ecológicos são os mais indicados para a avaliação dos déficits nas funções executivas, podendo auxiliar melhor no tratamento de pacientes com DA.

**Objetivo:**

O objetivo deste estudo preliminar é verificar o desempenho nas funções executivas usando a *Behavioural Assessment of the Dysexecutive Syndrome* - BADS em idosos saudáveis e pacientes com DA provável em fase inicial e a aplicabilidade desta bateria ecológica em nosso meio.

**Métodos:**

Avaliamos com a BADS 17 idosos saudáveis e 17 pacientes com DA provável em fase inicial, pareados em relação à idade, escolaridade e sexo.

**Resultados:**

Houve diferença estatisticamente significativa nos escores do MEEM e em medidas de funções executivas, memória e velocidade motora, também em alguns subtestes da BADS (Cartas-Troca de Regras, Seis elementos Modificado, Perfil Total, Padronizado, Padronizado pela idade e Classificação geral por idade) foram demonstradas diferenças entre os grupos. No questionário disexecutivo (DEX) da BADS de auto-avaliação e avaliação por outro se detectou diferença entre os grupos.

**Conclusões:**

A BADS mostrou-se eficaz na detecção dos déficits em funções executivas em nossa amostra, conforme confirmado pelos resultados dos demais testes que avaliam funções executivas.

Alzheimer’s disease (AD) is the most frequent cause of dementia in the world^1^ today where two surveys performed in Brazil showed a prevalence of 43.7 to 72.4% of all dementia cases.^2,3^

Although the main initial deficit is considered to be in the memory domain, an early impairment of executive functions^4,5^ is also found in AD and these deficits are correlated to functional impairment.^5-7^ The executive functions involve many components such as: volition, planning, purposive action, effective performance, as well as self-regulation and self-correction.^8^ Impairment in any of these areas causes a significant compromise in functional and behavioral aspects in humans. Executive deficits in AD patients can be associated with impairment in inhibition and co-ordination between processing and storing information.^5^ Additionally, some neuropsychiatric disturbances present in AD patients (anxiety, depression, apathy) are associated with a poorer performance in executive tasks.^9^ However, it is possible that executive impairments may not be present in all AD patients, due to neuropathological and clinical heterogeneity.^10^

Early detection of these deficits should allow better caregiver guidance by medical staff, as well as help the patient to learn internal and external patient strategies to achieve greater autonomy.

Formal evaluations of executive functions imply determination of activities by the examiner, regarding how and when to perform the task.^8^ For this reason it is important to investigate heterogeneity of executive deficits with “ecological tests”. These kinds of tests allow task execution without previous rules, imitating actual activities of daily living, and entail free organizing and execution of tasks.

The aim of this preliminary study is to verify performance in executive functions using the Behavioural Assessment of the Dysexecutive Syndrome – BADS^11^ in healthy elders and AD patients and to evaluate the applicability of this test in our environment.

## Methods

### Participants

We evaluated 17 probable mild AD patients according to criteria of the National Institute of Neurological and Communicative Disorders and Stroke, Alzheimer’s Disease and Related Disorders Association (NINCDS-ADRDA)^12^ who were followed in a specialized outpatient clinic of Hospital Santa Marcelina and Hospital São Paulo. They were matched for age, educational level (Table 1) and gender (χ^2^=0.11; p= 0.72) to 17 healthy controls that fulfilled the following inclusion criteria:

**Table 1:** Comparison among AD patients and controls for formal neuropsychological scores.

	Controls			AD patient			Mann-Whitney test
	Mean (SD)	µ		Mean (SD)	µ		p value
Age	71.4 (4.6)	71		72 (4.7)	72		0.70
Educational level (years)	9.9 (5.4)	11		9.8 (5.5)	11		0.90
MMSE	28.2 (1.4)	28		23.4 (2.1)	23		<0.01
Pfeffer	0	0		11.2 (6.8)	10		<0.01
GDS (15 items)	1.8 (1.8)	1		3.6 (1.8)	3		<0.01
Fist-palm-edge	2.1 (1.0)	2		1.3 (1.2)	1		0.07
Digit span forward	4.7 (1.1)	5		4.5 (1.0)	5		0.71
Digit span backward	3.7 (0.5)	4		3.0 (0.96)	3		0.04
Excluded Letter E	19.0 (4.8)	20		13.2 (4.2)	14		<0.01
Excluded Letter A	19.8 (3.4)	20		12.7 (4.5)	12		<0.01
Action Fluency	16.4 (4.1)	16		10.4 (4.1)	10		<0.01
Animal fluency	17 (3.8)	16		10.7 (2.5)	11		<0.01
TMT AA (errors)	0.1 (0.5)	0		0.1 (0.3)	0		0.80
TMT A (time)	56.2 (23.7)	47		95 (54.2)	70		<0.01
TMT B (errors)	0.8 (1.1)	0		1.9 (1.3)	2		0.02
TMT B (time)	180.0 (96.1)	156		225.0 (105.6)	196		0.10
BCSB -Naming	9.9 (0.24)	10		10.0 (0.0)	10		0.76
BCSB -Incidental	5.2 (1.2)	6		3.7 (1.9)	4		<0.01
BCSB -Immediate	7.8 (0.8)	8		5.7 (1.2)	6		<0.01
BCSB -Learning	8.7 (0.9)	9		5.9 (1.5)	7		<0.01
BCSB -Delayed recall	7.5 (1.5)	8		2.3 (2.5)	2		<0.01
BCSB -Recognition	10 (0)	10		7.8 (2.7)	9		<0.01
Stroop Test Part I (time)	19.8 (5.3)	19		30.7 (24.9)	23		0.02
Stroop Test Part I (errors)	0(0)	0		0 (0)	0		0.76
Stroop Test Part II (time)	27.6 (9.7)	27		35.6 (15.8)	33		0.07
Stroop Test Part II (errors)	0(0)	0		0(0)	0		1.00
Stroop Test Part III(time)	47.9 (16.2)	46		64.4 (26.3)	64		0.04
Stroop Test Part III (errors)	1.7 (2.2)	1		4.2 (6.1)	1		0.21
CLOX 1	13.4 (1.3)	14		12.0 (3.0)	13		0.09
CLOX 2	14.1 (0.7)	14		13.5 (1.1)	14		0.19

- Scores greater than or equal to the median score for educational level on the MMSE.^13,14^
- Functional Activities Questionnaire^15^, with scores less than 2 points.
- Scores on Geriatric Depression Scale below 6 points (15-item version)^16^
- Absence of psychiatric and/or neurological disorders.

Subjects from both groups were 60 years’ old or more, had an educational level of 4 years or more, with corrected visual or hearing deficits and, no motor impairment (rheumatologic or orthopedic causes).

The research protocol was submitted to and approved by the Ethics Committees of Santa Marcelina and São Paulo Hospitals. All controls and caregivers signed the informed consent prior to the interview.

### Neuropsychological formal evaluations

The neuropsychological formal evaluation was performed, including the following tests:

- *Digit span (forward and backward)*^17^ – evaluating attention and working memory, respectively.
- *Memory of figures of the Brief Cognitive Screening Battery* (BCSB)^18,19^ – with naming, visual perception, incidental memory, immediate memory, learning, delayed recall after 5 minutes, and recognition of 10 drawings/figures, with a maximum score of 10.
- *Trail making test* (TMT) – Parts A and B^20^, assessing selective attention, motor speed (part A), shifting ability, and inhibitory control (part B). Number of errors and timed of performance on each part was measured; the test was stopped after three consecutive errors or after 300 seconds.
- *Stroop test (Victoria version)*^21,22^ – Part I - naming the color of the rectangles as quickly as possible; part II – naming the color in which the words are printed, and Part III: naming the color of the ink in which the word is printed. This test evaluates inhibitory control, interference vulnerability, and shifting set. We scored the number of errors and time in performing each part.
- *Verbal fluency tests* – Animal category: generating as many animals as possible in 60 seconds^23^; action fluency: subjects were asked to generate as many different things that people do, (e.g. to eat, to wash)^24^; excluded letter fluency^25^ – the participant had to produce as many words as possible that did not contain a designated letter (in this case, the letter E after the letter A) during 60 seconds in each trial, we asked subjects to generate words not containing letter E, and then letter A. These tests evaluate inhibitory control, shifting set, thought organization, and speed of processing.
- *Luria's fist-edge-palm test*^26^ – The examiner performed a sequence of 3 dominant-hand positions and asked the subject to reproduce the sequence. The examiner demonstrates the positions up to 3 times. We scored 3 points for success in the first attempt, 2 points for success after 2 attempts and 1 point for success after 3 attempts. The subject scored zero if they were unable to perform the task correctly.
- *Clock design - CLOX*^27^ – With two parts: CLOX 1, patients are asked to draw a clock and put 13:45 h, verifying executive control; CLOX 2, patients are asked to copy a clock drawing, evaluating visuoconstruction ability.

### Ecological evaluation

- Behavioural Assessment of the Dysexecutive Syndrome - BADS^11^ composed of six sub-tests, with a maximum score of 24 points. An overall classification is obtained: impaired, borderline, low average, average, high average, superior, and very superior.

This battery of tests evaluates executive functions such as: inhibitory control and monitor behavior, planning, priorities, problem solving, and cognitive fl exibility.

- *Rule Shift Cards test* – There are 21 cards and patients in the first rule, must say “yes” for red cards and “no” for black ones and in the second rule must say “yes” if two sequential cards were the same color and “no” if colors were different. This rule, typed on a card, is left in full view (for the patient) throughout, to reduce memory constraints. Maximum score of 4.
- *Action Program test* – The subject is presented with a rectangular stand into one end of which, a large transparent beaker is placed with a removable lid having a small central hole in it. A thin transparent tube at the bottom of which is a small piece of cork is placed into the other end of the stand. The beaker is two thirds full of water. To the left of the stand, a metal rod is placed (roughly- L- shaped) which is not long enough to reach the cork, and a small screw top container on its side, with its top unscrewed and lying beside it. Subjects are asked to get the cork out of the tube using any of the objects in front of them but without lifting up the stand, the tube or the beaker and without touching the lid with their fingers. Maximum score of 4.
- *Key Search test* – Subjects are presented with an A4-sized piece of paper with a 100mm square in the middle and a small black dot 50mm below it. The subjects are told to imagine that the square is a large field in which they have lost their keys. They are asked to draw a line, starting on the black dot, to show where they would walk to search the field to make absolutely certain that they would find their keys. Maximum score of 4.
- *Temporal Judgment test* – 4 questions about common events which take from a few seconds to several years and subjects are asked to guess the time of each event (seconds, minutes, years). Maximum score of 4.
- *Zoo Map test* – Subjects are required to show how they would visit a series of designated locations on a map of a zoo. However, when planning the route certain rules must be obeyed (in our search these rules were left in front of participant as a memory cue). The map and rules have been constructed so that there are only four variations on a route that can be followed in order that none of the rules of the test are infringed. There are two trials. Maximum score of 4.
- *Adapted version of The Modified Six Elements test*28 – The subject has ten minutes to complete three different tasks (geometrical figures to be copied, picture naming and arithmetic), divided into two parts (A and B). The subjects are required to attempt at least something from each of the six sub tasks within the time allotted. They are not allowed to do two parts of the same task consecutively (e.g. naming and arithmetic of part B), and are advised to do a little of some part. The rules were left in front of subjects. This test measures how well subjects organize themselves. Maximum score of 4.

### Behavioral evaluation

- Neuropsychiatric Inventory - NPI,^29,30^ with a maximum score of 132 points.

Dysexecutive Questionnaire (DEX) of BADS, this is not used in the calculation of the profile score for the battery. This comprises a 20-item questionnaire constructed in order to examine the range of problems associated with the Dysexecutive syndrome. Two score forms were used: self-ratings performed by patient, and other - ratings fulfilled by caregiver, with a maximum score of 80 points.

*Mood evaluation* – Geriatric depression scale – GDS^31,16^, with 15 items. Scores above six suggest depression.

*Statistical analysis* – Statistical analyses were performed with nonparametric tests (Mann-Whitney, Chi-Square and Fisher exact test) with the Bio Estat 3.0 program.

## Results

There were no differences among patients and controls in relation to age (p=0.70), educational level (p=0.90) and gender (χ^2^=0.11; p=0.72). For both groups, GDS scores were below six points, so no depressive patients were included in our samples.

Table 1 shows the scores obtained by patients and healthy controls on neuropsychological formal evaluations. We observed a significant difference in all tests, except for Luria's fist-edge-palm test, digit span forward, Trail Making A (errors), Trail Making B (time), BCSP (naming), Stroop test: Part I (errors), Part II (time and errors) and Part III (time) and CLOX 1,2.

Table 2 shows the scores obtained by patients and healthy controls in Ecological evaluations. We observed a significant difference in all tests, except for BADS (Action Program test, Key Search test, Temporal Judgment test and Zoo Map test).

**Table 2:** Comparison among AD patients and controls for BADS scores.

	Controls			AD patient			Mann-Whitney test
	Mean (SD)	µ		Mean (SD)	µ		p value
Rule Shift Cards test	2.4 (1.2)	3		1.3 (1.3)	1		0.02
Action Program test	3.1 (1.3)	4		2.1 (1.6)	2		0.14
Key Search test	1.6 (1.1)	2		1.3 (1.4)	1		0.33
Temporal judgment test	1.8 (1)	2		2.3 (0.8)	2		0.11
Zoo map test	1.8 (1.4)	2		1.3 (1)	1		0.36
Six-elements test	3.6 (0.4)	4		2.1 (0.8)	2		<0.01
Total score	14.6 (3.6)	15		10.7 (4.4)	11		<0.01
Standard	83.3 (17.6)	85		64.2 (22)	67		<0.01
Standard by age	90.9 (19.2)	94		72 (22.3)	74		<0.01

The overall classification on BADS by patients and controls is described and compared in Table 3.

**Table 3:** Overall classification - BADS - comparison among patients and controls.

n (%)	I	B	LA	A	HA	S	VS	Total	
C	2 (11.7%)	3 (17.6%)	2 (11.7%)	8 (47.0%)	2 (11.7%)	0 (0.00%)	0 (0.00%)	17 (100%)	p=0.0214
AD	9 (52.9%)	4 (23.5%)	1 (5.88%)	2 (11.7%)	0 (0.00%)	1 (5.88%)	0 (0.00%)	17 (100%)	

C, controls; AD, AD patients; I, impaired; B, borderline; LA, low average; A, average; HA, high average; S, superior; VS, very superior. Fisher's Exact Test.

AD patients achieved a mean score of 18.7 points on the NPI (median value of 1). DEX – self ratings did not differ among controls and patients (p=0.79). However, we observed a significant difference among scores on DEX-self ratings and on DEX-other ratings among patients and their caregivers (p=0.04) as shown in Table 4.

**Table 4:** Comparison among patients and controls for DEX self-ratings and results of NPI in patients.

	Controls			Patients			Mann-Whitney Test
	Mean (S.D.)	µ		Mean (S.D.)	µ		p-value
DEX self-ratings	17.9 (7.7)	16		18.5 (12.9)#	20		0.79
DEX other ratings	N/A*			28.9 (16.7)#	26		
NPI	N/A*			18.7 (32.3)	1		

* N/A, Not applicable;

# Mann-Whitney test, p=0.04 (difference on DEX self and other ratings among patients).

## Discussion

Controls and patients differed on executive functions tests for: verbal fluency, TMT B (errors) and digit span backwards, showing impairment in shifting ability and inhibitory control in mild phases of AD. However, AD patients did not differ from controls in any part of the Stroop test, some slowing was demonstrated, which could aid inhibitory control (due to slowing) in these patients.

Some reports have shown a high sensitivity of these kinds of tests for dementia diagnosis and in initial executive impairment in mild AD.^25,27,33,39^

There was no difference on the CLOX 1, which evaluates executive function, in contrast with previous reports showing discrepant findings.^38^

We observed no difference among patients and controls on the fist-edge-palm test, where this test has been linked to activation of premotor and left parietal areas (adjacent to the intraparietal sulcus), cerebellum, contralateral sensoriomotor area, supplementary motor area and thalamus in functional studies.^36,37^ As our dementia group presented with mild stages we could expect this test to reveal a difference only in more severe degrees of dementia.

A decreased motor speed was observed in AD patients by time spent for execution of TMTA.

The performance of mild AD patients was poorer than controls in measures of incidental and immediate memory, learning, delayed recall and recognition (BCSB), as expected in AD from early stages.^38^ Decreased capacity in episodic memory can interfere in tasks involving executive functions.^4,28,39,40^ However, there was no correlation among memory and executive tests in our sample (data not shown). It is possible that these results could prove different with an increased number of patients. Moreover, it is important to note that the cards with rules were left in front of the subjects as mnemonic cues, and could have minimized memory influence on task performances.

The absence of differences observed between groups in naming, digit span forward and CLOX2 can be justified by the mild stage of AD.

There was no difference in four sub items of BADS (action programming, key search, temporal judgment, and zoo map), but there was a difference in total scores, standard, standard by age and overall classification by age, as well as, two sub-items (cards and six-elements). The number of participants was too low for adequate classification into seven groups, and one patient outperformed in overall classification by age, which did not occur in our control group. Increasing the number of participants may serve to blunt this effect. These findings suggest that some specific tests of BADS may be more sensitivity detecting executive deficits in our population.

Some reports with elders have verified poorer performance in the modified six-elements^41^ and zoo map,^42^ indicating impairment in planning by these subjects. A report of sixty-four subjects with acquired brain lesions has shown greater sensitivity in detecting planning deficits with modified six-elements and action programming.^43^ In Brazil, another report has demonstrated that “modified six elements” was better than other formal executive measures, confirming its sensitivity in detecting planning impairments and in qualitative investigation strategies.^28^

Increasing our casuistic we could establish correlations among behavioural disturbances and executive functions, as well as between DEX self-ratings and DEX other-ratings. Total scores of DEX self - ratings on BADS did not differ among controls and AD patients, although AD patients reported more executive complaints. We were able to observe a lack of awareness by patients of their difficulties, as AD patients scored differently to their caregivers on the DEX other-ratings scores.

BADS was efficient in detecting executive impairments in our sample, confirming results obtained by the other executive tasks performed.

Transcultural adaptations should be performed to prove real power to evaluate dysexecutive subjects, while almost all cognitive tests, including executive functions tests,^35^ are influenced by educational level. Our control group had 11 years of median schooling, yet 47% were classified as average, a finding which may be related to particular cultural influences. Further studies are necessary in order to confirm these findings.
